# Balance Assessment Under Different Conditions in Patients with Surgically Treated Pilon Fracture Compared to Healthy Controls: A Pilot Study

**DOI:** 10.3390/life15081319

**Published:** 2025-08-20

**Authors:** Andrei-Daniel Bolovan, Gheorghe-Bogdan Hogea, Elena-Constanta Amaricai, Alexandra-Roxana Tapardea, Alina-Daniela Totorean, Anca-Raluca Dinu, Adrian-Emil Lazarescu, Mihai-Alexandru Sandesc, Jenel-Marian Patrascu

**Affiliations:** 1 Doctoral School, “Victor Babes” University of Medicine and Pharmacy, 300041 Timisoara, Romania; andrei.bolovan@umft.ro (A.-D.B.); roxana.tapardea@umft.ro (A.-R.T.); 2 Research Center for Assessment of Human Motion, Functionality and Disability, Department of Rehabilitation, Physical Medicine and Rheumatology, “Victor Babes” University of Medicine and Pharmacy, 300041 Timisoara, Romania; totorean.alina@umft.ro (A.-D.T.); dinu.anca@umft.ro (A.-R.D.); 3 Department of Orthopedics and Traumatology, “Victor Babes” University of Medicine and Pharmacy Timisoara, 300041 Timisoara, Romania; hogea.bogdan@umft.ro (G.-B.H.); lazarescu.adrian@umft.ro (A.-E.L.); sandesc.mihai@umft.ro (M.-A.S.); jenel.patrascu@umft.ro (J.-M.P.J.); 4 “Pius Brinzeu” Emergency Clinical County Hospital, Bld Liviu Rebreanu, No. 156, 300723 Timisoara, Romania; 5 Research Center Teodor Sora, Department of Orthopedics II, “Victor Babes” University of Medicine and Pharmacy, Eftimie Murgu Square, No. 2, 300041 Timisoara, Romania

**Keywords:** pilon fractures, static balance, dynamic balance, force plates, sensorimotor rehabilitation

## Abstract

Background: Tibial pilon fractures are usually high-energy fractures that are linked to higher rates of complications and poor clinical outcomes, particularly concerning pain and walking impairments. However, few studies have evaluated postural stability among patients surgically treated for pilon fractures. Objective: This pilot study aimed to evaluate static and dynamic balance in patients who have undergone unilateral pilon fracture fixation, compared to matched healthy controls. Methods: Ten adult patients, post-fixation for unilateral pilon fracture (with clinical and radiological evidence of fracture healing and ability to bear full weight on the affected lower limb), completed a series of balance tests on K-Force plates. Ten matched healthy controls performed the same tests. Outcomes included CoP path length, CoP mean velocity, time to stabilisation (TTS), and peak force normalized to body weight. Results: Patients showed significantly increased mediolateral sway during bipodal stance (mean CoP velocity of 33.7 vs. 22.4 mm/s, *p* < 0.01), especially under eyes-closed conditions. In single-leg stance, CoP velocity on the affected limb was more than double that of controls (118 vs. 54 mm/s, *p* = 0.036). Dynamic tests revealed longer TTS after landing (1563 vs. 501 ms, *p* = 0.048) and lower force output during squats. The unaffected limb performed nearly normally in static tasks but was slower during dynamic stabilization. Conclusions: Even after fracture healing, patients with tibial pilon fractures show persistent sensorimotor deficits and impaired balance.

## 1. Introduction

Pilon fractures are relatively rare, resulting in higher complication rates and poor clinical outcomes, particularly regarding walking impairments [1,2].

In clinical practice, various methods are used to stabilize pilon fractures. Treatment commonly involves open reduction and internal fixation. These fractures are primarily caused by high-energy trauma and are often associated with soft tissue injuries. Typically, pilon fractures require multiple surgeries for effective management, resulting in increased costs and prolonged recovery periods [1,3]. After sustaining a tibial pilon fracture, individuals may face long-term complications. This is especially true for comminuted intra-articular fractures resulting from high-energy trauma. Research has shown that patients with tibial pilon fractures score lower on health-related quality of life questionnaires compared to uninjured individuals of the same age, as well as people with chronic illnesses [4].

In many cases, the function of the affected ankle is not fully restored, severely impacting the quality of life for those affected [4,5]. Patients who undergo surgery for this type of fracture report significant loss of ankle joint function and experience daily pain.

Most publications discuss treatment techniques for pilon fractures and evaluate their clinical and radiological outcomes. However, only a few studies assess functional outcomes, such as balance and ankle stability [1,3,4].

Posttraumatic arthrosis frequently complicates tibial pilon fractures, and studies indicate that clinical outcomes can worsen over time. Injuries to soft tissues, prolonged immobilization, and extended periods of non-weight-bearing on the injured side due to tibial pilon fractures can result in muscle atrophy, decreased strength, and balance issues [6,7,8].

Ankle fractures can lead to balance disturbances, significantly impacting walking and functional mobility [8].

Postural balance involves maintaining a specific posture in response to external disturbances. Therefore, clinical assessments are essential for identifying particular issues that could lead to falls, injuries, and instability in the ankle joint [9].

Postural balance tests serve as practical tools for monitoring ankle instability in patients [10].

Postural control is assessed through static and dynamic balance tests conducted during bipodal and unipodal stance. These assessments can be performed objectively using force platforms [11]. Utilizing center of pressure (CoP)-derived parameters for balance evaluation has proven effective in estimating fall risk and joint instability [12]. These CoP parameters, which are quantified and analyzed through force platforms [11], are considered a gold standard for measuring balance [13].

To differentiate bipodal balance between patients and healthy individuals with their eyes open and closed, a range of CoP trajectory variables can be used, both in the time domain (such as mean velocity, distance, or area) and the frequency domain (including total power and median frequency). Notably, the mean CoP velocity has been shown to be the most effective measure for detecting changes and differences across eye conditions in the two groups [12,14].

Although the Single-Leg Balance test is widely used to predict functional ankle instability and assess the risk of sprains or falls [15], there is a lack of studies focusing on specific CoP parameters for analyzing unipodal postural control using force platforms. Interestingly, the CoP mean velocity stands out as a reliable stabilometric measure for distinguishing patients with a history of ankle sprains from healthy individuals during single-leg balance tests with eyes closed [15].

To maintain balance, the central nervous system integrates visual, vestibular, and proprioceptive information to generate motor commands that activate muscles in coordinated patterns [16].

Ankle proprioception plays a crucial role in balance control. However, maintaining stability during a single-leg stance also depends on coordinated efforts from the hip, knee, and trunk muscles, along with psychological and contextual factors [17,18]. Both balance control and ankle proprioception are negatively related to injured ankles [17]. The literature also reports a connection between ankle proprioception and the risk of ankle injury. Persistent, impaired ankle proprioception after an injury can lead to long-term declines in postural and balance control [19].

Portable force plates can indirectly assess ankle proprioception through balance and sway analysis. Single-leg stance or eyes-closed balance tests on these plates can help infer ankle proprioception, as increased sway or instability often suggests sensorimotor deficits [20,21,22].

The objective of the current research was to compare both the static and dynamic balance parameters in patients who had undergone pilon fracture surgery and in healthy subjects. Our study hypothesized that the static and dynamic balance would be impaired in the above-mentioned category of patients when compared to controls.

## 2. Materials and Methods

### 2.1. Participants

In total, 15 patients with surgically treated unilateral tibial pilon fractures were asked to participate in the study. The inclusion criteria were clinical and radiological evidence of fracture healing, ability to bear full weight on the affected lower limb, and the affected lower limb being the dominant one. We assessed pain levels using the pain domain of the 36-Item Short Form Survey (SF-36) [23]. All patients reported scores corresponding to either no pain or mild pain, indicating minimal pain at the time of testing. Individuals were excluded from the study if they had a history of trauma or fractures in the affected lower limb or the opposite lower limb, or if they had neurological or other health conditions that could have impaired walking or altered muscle function. Those with lower leg asymmetry not caused by the tibial pilon fracture were also excluded. Additionally, patients with psychiatric disorders, severe cardiovascular disease, morbid obesity (BMI > 40), or cancers were excluded due to the risks these conditions pose to compliance and follow-up.

Five patients met the exclusion criteria: lumbar disc hernia (2), stroke (1), prostatic cancer (1), and depression (1). Additionally, 10 gender- and age-matched healthy volunteers were also recruited as controls. Participation in the study was voluntary, and written informed consent was obtained from all participants. The study was approved by the Ethics Committee of “Victor Babes” University of Medicine and Pharmacy in Timisoara, Romania, and was conducted in accordance with the Helsinki Declaration. Clinical characteristics of the patients and controls, including age, height, weight, and body mass index, were recorded. The two groups were similar regarding anthropological features (Table 1). All patients and controls were right-leg dominant.

### 2.2. Assessment

The static and dynamic balance were assessed using K-Force plates (Kinvent, Montpellier, France [24]). The data were collected via a Bluetooth connection with the Kinvent Physio application and stored on a Samsung tablet. Before the actual assessment, the subjects were familiarized with the force platforms. The study protocol involved a single session testing (static and dynamic balance testing) for both the patient group and the control group. Static balance was evaluated under three conditions: bipodal stance with eyes open, bipodal stance with eyes closed, and single-leg balance (on the right leg, followed by the left). Dynamic balance was assessed during a single-leg landing (on the right leg, followed by the left) and during a squat analysis.

When performing the bipodal test with eyes open, the participant stood upright on the platform, with their arms at their sides, and focused on a marker located 3 m away. The participant remained motionless throughout the entire test. Each session lasted 30 s, with a 10 s rest between repetitions; three repetitions were performed for each condition. The procedure was then repeated with eyes closed. For the single-leg balance test, the subject placed the foot of the tested leg in the center of the platform, with hands placed on the hips and the non-weight-bearing leg slightly flexed at the hip and knee. The participant focused on a marker 3 m away, remaining still during the test. Each trial lasted 10 s, followed by a 10 s rest, with three repetitions conducted for each condition. The following parameters were recorded for each static balance condition (bipodal stance with eyes open, bipodal stance with eyes closed, right single-leg balance, and left single-leg balance): center of pressure (CoP) path length (mm), ellipse area (mm^2^), and CoP mean velocity (mm/s).

Following the static balance assessment, dynamic balance was evaluated through single-leg landing and squat analysis. The single-leg landing is a unipodal dynamic balance test. The participant stepped onto a 19 cm box, with hands placed on the hips. The participant took a step forward with the test leg, dropping from the step and landing solely on the force platform. The untested leg must leave the step at the same time the test leg contacts the platform. After landing, the participant must stabilize as quickly as possible, keeping hands on hips and eyes focused on the marker set 3 m away. The participant must hold this position for 10 s. Three repetitions were performed for each test, with 10 s of rest between repetitions. The following parameters were recorded: ellipse area (mm^2^), time to stabilization (ms), and peak force normalized to body weight (kg/kg).

During the squat analysis, the participant stood upright, then descended into knee flexion of 90–100°, and then returned to the starting position. Each test lasted 20 s, with a 10 s rest between repetitions; three repetitions were performed for each test. The recorded parameters included: total CoP displacement (mm), ellipse area (mm^2^), mean CoP velocity (mm/s), and peak force normalized to body weight for both the right and left leg (kg/kg). CoP path length reflects the total length (in millimeters) of the center of gravity shift during the test. Ellipse area indicates the size (in mm^2^) of the ellipse containing all the measured center of gravity points plotted on a Cartesian coordinate system. Total CoP displacement measures the distance the CoP moves in the anteroposterior and lateral directions from a reference point or position [25,26].

CoP mean velocity (measured in millimeters per second) is the average speed at which the CoP moves during the assessment. For a consistent sampling interval, the mean velocity is calculated as the sum of the distances between consecutive points divided by the total recording time [12].

Time to stabilization is the time it takes for a subject to return to a stable state following a jump [27].

Peak force normalized to body weight (measured in kg/kg) reflects the maximum force a person can generate relative to their body weight, often measured during activities like jumping or weightlifting. This is typically expressed as a ratio (peak force/body weight) [28].

For each of the testing conditions, we retained the average values of the three repetitions.

### 2.3. Statistical Analysis

All statistical analyses were conducted using GraphPad Prism 5.0 for Windows. Descriptive statistics, including mean and standard deviation, were calculated for all variables. Comparisons of the stabilometry parameters (for both static and dynamic balance assessments) between patients and controls were made using Student’s unpaired *t*-test or a Chi-squared test. A *p*-value less than 0.05 was considered statistically significant [29].

## 3. Results

The results of stabilometry assessment in bipodal stance (with eyes open and with eyes closed) are shown in Table 2 and Table 3. In both testing conditions, CoP path length and CoP mean velocity were significantly higher in the patient group compared to healthy controls. Although the ellipse area had increased values in patients, there were no statistically significant differences between the two groups.

When assessing single-leg balance, we compared the stabilometry parameters between the two groups for the right and left legs, respectively (Table 4). For the single-leg balance on the right leg (the affected leg in patients and the dominant leg in controls), patients had significantly higher values of CoP path length and CoP mean velocity compared to controls. For the single-leg balance on the left leg (non-affected leg in patients and non-dominant leg in controls), no significant differences were observed in CoP path length, ellipse area, or CoP mean velocity.

When comparing the single-leg landing parameters of patients and controls, we found no significant differences in ellipse area and peak force normalized to body weight for either the right or left legs (Table 5). However, the time to stabilization during landing on the right and left legs was significantly longer in the patient group. The patients took more time to stabilize, regardless of whether they landed on the affected or non-affected leg.

Table 6 presents the data from the squat analysis. During the 20 s squats, there were no significant differences in CoP total displacement, ellipse area, and CoP mean velocity between patients and controls. The peak force normalized to body weight on the right leg was significantly lower in patients (with the right leg being the affected one) compared to controls (where the right leg is the dominant one). For the left leg, which was non-affected in patients, there were no differences in peak force normalized to body weight between patients and controls (the left leg being the non-dominant one).

## 4. Discussion

This pilot study analyzes both static and dynamic postoperative balance in adults following unilateral tibial pilon fracture fixation, using instrumented K-Force plates. Nearly all the static conditions showed that patients had significantly greater mediolateral sway, as indicated by CoP path lengths and higher mean CoP velocities, compared to the matched controls. In eyes-open bipodal standing, CoP path length and velocity were about 60% higher in patients (1015 mm vs. 638 mm and 33.7 mm/s vs. 22.4 mm/s, respectively). In eyes-closed bipodal standing, the difference between groups increased, with patient mean velocity reaching 36.4 mm/s, suggesting that reliance on vision alone is insufficient to restore standard postural control after fracture healing.

During single-leg stance, the deficit was side-specific. On the fractured leg, patients’ CoP velocity was more than double that of the controls (118 mm/s vs. 54 mm/s, *p* = 0.036), while their non-affected leg behaved similarly to control non-dominant limbs. This suggests that, despite radiological union and full weight-bearing clearance, the fractured leg shows delayed neuromuscular responses, whereas the healthy opposite side remains largely unaffected.

The dynamic balance revealed a different limitation. Peak ground-reaction forces during single-leg landings were comparable between groups, indicating that patients were capable of bearing the load; however, the time to stabilization (TTS) was nearly three times longer (e.g., 1563 ms vs. 501 ms on the affected side, *p* = 0.048). Longer TTS indicates delayed sensorimotor integration rather than weakness. This is supported by the squat analysis, where the fractured leg (right) produced significantly lower peak force compared to the controls (0.625 × vs. 0.843, *p* = 0.0009), despite no significant differences in total CoP displacement, ellipse area, and mean CoP velocity between patients and controls.

The reliability of the K-Force plates used in the current study has been independently verified [11,24]. The mean CoP velocity and time to stabilization (TTS) measured with these plates closely align with the results obtained from a multi-axis laboratory force platform [11]. Therefore, the significant effects observed between groups are unlikely to be artifacts of the hardware. The study by Serrano et al. [11] investigated the validity and reliability of K-Force plates in assessing the unipodal balance of patients and reported excellent test–retest reliability for CoP mean velocity and time to stabilization during single-leg stance tasks, with ICC values over 0.75. It demonstrated that K-Force plates can serve as an alternative to multi-axis force platforms for assessing bipodal or unipodal static and dynamic balance.

Due to their user-friendliness, lightweight design, and portability, the K-Force plates are suitable for field use or clinical settings, enabling semi-automatic quantitative diagnostics that can guide individualized rehabilitation of the lower limb.

Research on postural control after pilon fractures is limited; however, our findings align with broader studies on chronic ankle instability (CAI), which report similar postural impairments. A systematic review and meta-analysis by Xue et al. [21] showed that individuals with CAI have significantly higher mean COP velocities in both anterior–posterior and mediolateral directions during static single-leg stance. Hong et al. [30] confirmed these findings in their study with dancers and non-dancers, showing that CAI patients have an increased ellipse area and mean CoP velocities, especially when eyes are closed. Wikström et al. [31] also identified deficits in dynamic balance using time-to-stabilization metrics, which supports the CoP velocity results and highlights ongoing neuromuscular issues in individuals with ankle instability. In our research, we observed that the mean CoP velocity during single-leg stance on the affected limb is notably higher than typical values reported in the literature, around 70 mm/s for CAI patients and 40 mm/s for healthy individuals. This higher velocity likely results from the combined impact of the fracture and surgical treatment, leading to lasting sensorimotor deficits and postural instability. The results of our dynamic balance tests showed a longer TTS and decreased force output, consistent with the balance deficits described by Wikström et al. [31], and suggest that patients recovering from pilon fractures experience postural instability comparable to that seen in CAI populations, highlighting the need for rehabilitation that targets both static and dynamic balance. The results of Guo et al. [32] have shown that balance training is beneficial in improving daily living and sports abilities of CAI patients, as well as enhancing the dynamic stability of the ankle joint on the posterior side. Despite advancements, there is still inconsistency in the training methods, intensity, frequency, and duration of balance training, and a standardized, specific balance training program is still lacking. The development of a more targeted balance training program could be a key area for future research.

This study has several limitations that may affect the interpretation of the results. The small sample size (n = 10 patients) limits the extent to which the findings can be applied. As a pilot study, these results should be seen as preliminary, with larger groups needed to verify them. The observed impairments in balance and postural control—increased CoP path length and mean velocity during static tasks, longer TTS after landing, and reduced peak force during squats—should be interpreted with caution. The small participant number prevents subgroup analyses and controlling for factors such as time since surgery, rehabilitation protocols, or injury severity, all of which can affect neuromuscular outcomes. Also, our dynamic tests revealed longer TTS and lower force in the affected limb. Still, these issues may reflect broader sensorimotor adaptations to injury and surgery that were not fully captured in our study. A larger sample could enable more definitive conclusions about the consistency and clinical importance of these findings.

## 5. Conclusions

In patients with surgically treated pilon fractures, static balance tests under different conditions (bipodal stance with eyes open, bipodal stance with eyes closed, and single-leg balance) revealed poorer postural stability compared to healthy controls. However, no significant differences were observed when assessing the dynamic balance parameters during single-leg landing and squat analysis, except for the time to stabilization. When compared to controls, patients required more time to stabilize when landing on the affected leg and the non-affected leg, respectively.

## Figures and Tables

**Table 1:** Characteristics of patients and controls.

	Patients (n = 10)	Controls (n = 10)	*p*
Age (years), mean (SD)	43.9 (9.8)	43.5 (7.7)	0.92
Gender			
Males (n)	9	9	
Females (n)	1	1	
Height (cm), mean (SD)	178.9 (7.3)	175.7 (9.8)	0.65
Weight (kg), mean (SD)	97.7 (19.7)	89.4 (7.2)	0.06
BMI (kg/m^2^), mean (SD)	31.7 (5.7)	29.8 (6.6)	0.057

SD: standard deviation; n: number of subjects; BMI: body mass index.

**Table 2:** Stabilometry parameters in bipodal stance (eyes open) in patients and controls.

	Patients (n = 10)	Controls (n = 10)	*p*
CoP path length (mm) (mean ± SD)	1015 ± 69	637.9 ± 69	**0.0012**
Ellipse area (mm^2^) (mean ± SD)	352 ± 198	286.1 ± 79.5	0.76
CoP mean velocity (mm/s) (mean ± SD)	33.7 ± 2.2	22.4 ± 1.8	**0.0013**

CoP: center of pressure; SD: standard deviation; n: number of subjects. The bolded *p*-values indicate significant differences.

**Table 3:** Stabilometry parameters in bipodal stance (eyes closed) in patients and controls.

	Patients (n = 10)	Controls (n = 10)	*p*
CoP path length (mm) (mean ± SD)	1095 ± 53.3	634.2 ± 53	**<0.0001**
Ellipse area (mm^2^) (mean ± SD)	712.7 ± 449.9	224.4 ± 101.8	0.30
CoP mean velocity (mm/s) (mean ± SD)	36.4 ± 1.8	22.58 ± 1.4	**<0.0001**

CoP: center of pressure; SD: standard deviation; n: number of subjects. The bolded *p*-values indicate significant differences.

**Table 4:** Single-leg balance parameters in patients and controls.

	Patients (n = 10)		Controls (n = 10)		*p* ^a^	*p* ^b^
	Right Leg	Left Leg	Right Leg	Left Leg		
CoP path length (mm) (mean ± SD)	1189 ± 403.2	777.3 ± 100	545.2 ± 25.9	648.5 ± 105.4	**0.037**	0.21
Ellipse area (mm^2^) (mean ± SD)	4095 ± 2194	1512 ± 297	1156 ± 86.4	1706 ± 473	0.26	0.30
CoP mean velocity (mm/s) (mean ± SD)	118 ± 39.9	77.2 ± 9.8	54.1 ± 2.5	64.27 ± 10.4	**0.036**	0.20

CoP: center of pressure; SD: standard deviation; n: number of subjects; *p* ^a^ relates to the difference between the right leg parameters of the patients and controls; *p* ^b^ relates to the difference between the left leg parameters of the patients and controls. The bolded *p*-values indicate significant differences.

**Table 5:** Single-leg landing parameters in patients and controls.

	Patients (n = 10)		Controls (n = 10)		*p* ^a^	*p* ^b^
	Right Leg	Left Leg	Right Leg	Left Leg		
Ellipse area (mm^2^) (mean ± SD)	2423 ± 1071	2855 ± 1784	1124 ± 200.6	1599 ± 368.2	0.24	0.49
Time to stabilization (ms) (mean ± SD)	1563 ± 490	1039 ± 127	501 ± 105	618 ± 132	**0.048**	**0.034**
Peak force normalized to body weight (kg/kg) (mean ± SD)	1.074 ± 0.028	1.232 ± 0.053	1.24 ± 0.11	1.256 ± 0.10	0.18	0.84

SD: standard deviation; n: number of subjects; *p* ^a^ relates to the difference between the right leg parameters of the patients and controls; *p* ^b^ relates to the difference between the left leg parameters of the patients and controls. The bolded *p*-values indicate significant differences.

**Table 6:** Squats analysis in patients and controls.

	Patients (n = 10)	Controls (n = 10)	*p*
CoP total displacement (mm) (mean ± SD)	4303 ± 564.4	3221 ± 223.8	0.091
Ellipse area (mm^2^) (mean ± SD)	9418 ± 1309	8454 ± 1019	0.56
CoP mean velocity (mm/s) (mean ± SD)	214.8 ± 28.1	160.8 ± 11.15	0.09
Peak force normalized to body weight on the right leg (kg/kg) (mean ± SD)	0.625 ± 0.03	0.843 ± 0.045	**0.0009**
Peak force normalized to body weight on the left leg (kg/kg) (mean ± SD)	0.741 ± 0.028	0.800 ± 0.037	0.22

CoP: center of pressure; SD: standard deviation; n: number of subjects. The bolded *p*-value indicates a significant difference.

## Data Availability

The data presented in this study are available upon request from the corresponding author (E.C.A).

## Abbreviations

The following abbreviations are used in this manuscript:

CoP	Center of Pressure
TTS	Time to Stabilization
CAI	Chronic Ankle Instability
SD	Standard Deviation
